# When Professional Networking Becomes Exhausting: Passive LinkedIn Use, Communication Overload, and Social Media Fatigue

**DOI:** 10.3390/bs16091512

**Published:** 2026-08-27

**Authors:** Phillip Ozimek, Annemarie Helga Spannenkrebs, Eileen Bohl, Elke Rohmann, Denise Hoffmann, Hans-Werner Bierhoff

**Affiliations:** 1 Department of Humanities, Vinzenz Pallotti University, 56179 Vallendar, Germany; 2 Department of Psychology, Ruhr University Bochum, 44801 Bochum, Germany

**Keywords:** professional social media, social media fatigue, communication overload, information overload, LinkedIn

## Abstract

Professional social networking has received considerably less research attention than private social media, particularly with regard to passive use and social media fatigue. The present study examined the association between passive LinkedIn use and social media fatigue and investigated whether communication overload and information overload may account for this association. In addition, the study provides a preliminary LinkedIn-specific adaptation of an existing measure of professional social media use, the LinkedIn Activity Questionnaire (LAQ). Data were collected from an online sample of 137 LinkedIn users. Correlation analyses and a parallel mediation model were conducted, controlling for weekly LinkedIn use. Passive LinkedIn use was positively associated with communication overload but not with information overload or social media fatigue. Both communication overload and information overload were positively associated with social media fatigue. The parallel mediation analysis revealed a significant specific indirect effect through communication overload, whereas the indirect effect through information overload was not significant. Neither the total nor the direct association between passive LinkedIn use and social media fatigue was significant. These findings suggest that communication overload may represent a particularly relevant pathway linking passive LinkedIn use and social media fatigue in professional social networking contexts. The findings also provide initial support for the usefulness of the LAQ as a LinkedIn-specific operationalization, while further psychometric validation is warranted.

## 1. Introduction

The use of social networking sites (SNSs) and how they influence the human psyche are topics that are increasingly being addressed in current research (Koh et al., 2024; Stangl et al., 2023). SNSs constitute a comprehensive subcategory of social media. While social media serves the purpose of creating and sharing content, SNSs focus on establishing relationships between users and facilitating the maintenance of these relationships (Matveev, 2024). SNSs have become indispensable today. They function as a means of communication and as platforms for the exchange of information (S. Kim et al., 2024).

Previous studies indicated that the influence of SNSs was both positive and negative depending on the variable considered (Koh et al., 2024). They exerted a positive impact by increasing satisfaction through establishing and maintaining emotional connections with friends as well as reducing feelings of loneliness, thereby improving social and psychological well-being (Mahapatra & Schatz, 2015; Brown et al., 2021; Dodemaide et al., 2021; Barker, 2009; Chan, 2018; Reimann et al., 2023). However, they exerted also a negative impact on well-being. For example, they elicited fear of missing out (FoMO) and, in general, fostered anxiety symptoms and depressive tendencies (Ozimek & Bierhoff, 2019; Brandenberg et al., 2019; David & Roberts, 2023; Erliksson et al., 2020).

The way social media is used is likely to influence whether positive or negative consequences follow. In this context, a distinction has been drawn between active and passive use, respectively. Passive use refers to consuming content without social interaction (Frison & Eggermont, 2015). Active use, on the other hand, implies that individuals interact with other users (Aikins, 2021). Verduyn et al. (2015) demonstrated that passive Facebook use negatively affects emotional well-being. Furthermore, the study by Escobar-Viera et al. (2018) revealed a positive association between passive use and depression, while active use was in contrast associated with lower levels of depression.

Another negative consequence that can arise from the use of SNSs, and which has received increasing attention from researchers in recent years, is the experience of social media fatigue (Jabeen et al., 2023; Świątek et al., 2021). Ravindran et al. (2014, p. 2317) define social media fatigue as “a subjective, multidimensional user experience that encompasses feelings such as tiredness, irritation, anger, disappointment, reluctance, loss of interest, or reduced need/motivation in connection with various aspects of social media use and interactions.” Several studies link social media fatigue to the cognitive overload that can result from the use of SNSs. The sheer volume of information and messages transmitted by SNSs can thus lead to an inability to cope with them and, consequently, to social media fatigue (Ravindran et al., 2014; Bright et al., 2015; Cao & Sun, 2017; K. Li et al., 2024).

Two key types of overloads are information overload and communication overload (Lee et al., 2016). Information overload describes a state in which users are confronted with more information than they can process (Eppler & Mengis, 2004; Ravindran et al., 2014). Communication overload, on the other hand, occurs when SNSs exceed the communication capacity of the respective user (Cho et al., 2011).

In addition to this cognitive level, social media fatigue also manifests on an emotional level, causing users to experience, for example, indifference, boredom, or a lack of interest when using SNSs (Yamakami, 2012; K. Lin, 2015; S. Zhang et al., 2016). Social media fatigue also often leads to a decrease in social media use, which manifests itself at the behavioral level. This behavior is divided into three types of usage intent: reduced frequency of use, brief breaks and leaving the current social network or switching to another one.

Current research on the nature of social media use, its resulting psychological effects, and the phenomenon of social media fatigue has primarily focused on private SNSs (Y. Zhang & Leung, 2014; Stangl et al., 2023; Koh et al., 2024). However, the use of professional SNSs, such as XING or LinkedIn, has also gained increasing attention in recent years (Utz & Breuer, 2016). While XING is widespread in Germany, Switzerland, and Austria, LinkedIn is used on a global scale (Thattil, 2023). LinkedIn currently has more than one billion members worldwide, making it the largest global professional SNS (LinkedIn, 2025). For this reason, and because LinkedIn places a stronger focus on the flow of information and exchange (Rätze, 2023), LinkedIn is the platform on which our research is focused.

Despite its growing relevance, empirical research on LinkedIn remains comparatively limited, particularly with regard to platform-specific behavioral assessment tools. Existing measures often fail to distinguish between professional and private SNS use or between different forms of LinkedIn engagement. There is also evidence that the use of professional SNSs can have negative consequences, such as higher depressive tendencies (cf. Ozimek & Bierhoff, 2019).

However, many studies focus primarily on private SNSs such as Facebook, Instagram, and WeChat, or address social media, in general, without concentrating on a specific platform (Baj-Rogowska, 2023). This disparity highlights the prevailing research gap regarding the focus on LinkedIn and how passive use, as well as resulting overload from usage, influences social media fatigue. Furthermore, passive use in the context of social media fatigue has often been addressed as an outcome rather than a predictor (Zhu & Bao, 2018; Tian et al., 2024). In addition, existing research lacks standardized behavioral measures specifically designed to assess LinkedIn use patterns, particularly passive LinkedIn use. Most prior studies have relied on generic social media measures or platform-unspecific self-reports, limiting the precise investigation of professional SNS behavior. Generic social media measures may also overlook behaviors that are particularly relevant in professional networking contexts. LinkedIn users may, for example, browse professional profiles, monitor profile visitors and profile statistics, search for professional contacts, follow companies, or engage with career-related content and networking opportunities. Such activities reflect professional networking, career management, and self-monitoring behaviors that are not necessarily captured by measures developed for general or private social media use. Consequently, a platform-specific measure may be useful for distinguishing different forms of LinkedIn engagement, particularly non-interactive browsing and monitoring behaviors. To address this methodological weakness, the present study introduces the LinkedIn Activity Questionnaire (LAQ) as a LinkedIn-specific adaptation of an existing measure of professional social media use. Accordingly, the present study contributes to the literature in two ways. First, it examines the relationship between passive LinkedIn use, overload, and social media fatigue within the context of professional SNSs. Second, it introduces the LinkedIn Activity Questionnaire (LAQ) as a platform-specific instrument for assessing LinkedIn usage behavior. The central research question is: Is passive LinkedIn use associated with social media fatigue through communication overload and information overload? The paper begins with a review of the current state of research and an explanation of key concepts. Based on this review, hypotheses are then derived and tested. Finally, the results are presented, interpreted, and discussed.

## 2. Theoretical Background

The theoretical analysis focuses on the LinkedIn platform and the relationship between passive use of SNSs and social media fatigue, as well as the problematic effects of communication overload and information overload.

### 2.1. LinkedIn

The LinkedIn platform was founded in 2002 and launched the following year (Shepherd, 2025). LinkedIn is the largest global professional network, with members in over 200 countries and regions (Shacklett & Hanna, 2025). The platform’s goal is to connect professionals worldwide, thereby increasing their productivity and success. To this end, LinkedIn offers opportunities to network with other users, promote oneself or one’s company, and publish content (Shepherd, 2025). Using the platform offers benefits for one’s professional career and has thus become a key tool for employees and recruiters (Bala, 2024). In addition to the benefits, research has also identified negative consequences of its use; for example, increased frequency of LinkedIn use has been associated with an increase in anxiety and depression (Jones et al., 2016). A study by Johnson and Leo (2020), which examined the relationship between LinkedIn use, ego depletion (i.e., mental exhaustion), and job searching via LinkedIn, found that the likelihood of a person feeling drained increases with the frequency of their LinkedIn use, while the likelihood of a successful job search decreases. These findings suggest that LinkedIn use can also have negative consequences and lead to a feeling of exhaustion.

To date, there has been little research on the phenomenon of social media fatigue that focuses exclusively on LinkedIn use. Only blog posts or posts on LinkedIn mention *LinkedIn fatigue* and suggest that users may suffer from it (McIntosh, 2021). However, there is hardly any reputable research on the topic, which is why this paper addresses it from a scientific perspective. The following section focuses on the central constructs of this research: social media fatigue.

### 2.2. Social Media Fatigue

Using social media can lead to increased fatigue. The cognitive effort required to actively engage with social media can deplete a person’s energy, leading to social media fatigue which describes the subjective feeling of tiredness and burnout resulting from social media activities (Ravindran et al., 2014). The experience of fatigue varies from person to person, as the same situation may lead to a mild feeling of tiredness in one person, while in another it may already result in a state of exhaustion (Ravindran et al., 2014). Social media fatigue is thus the self-reported subjective feeling of fatigue resulting from the use of SNSs. It is multidimensional, as the sensation can involve emotional, cognitive, and behavioral aspects, as noted by Ravindran et al. (2013). These three dimensions are described next.

When people become overwhelmed by their use of social media, for example, through constant replying or additional external demands, this can manifest on a cognitive level as impaired reasoning, memory, and thinking. This is evident, for example, in a decline in academic performance or in making mistakes. Health issues may arise on an emotional and psychological level, such as depression, frustration, burnout, or anxiety. Finally, on a behavioral level, various reactions may occur, such as ceasing use or reducing engagement in interactions.

The likelihood that users will stop using the service increases with the severity of their fatigue (Maier et al., 2015; Xie & Tsai, 2020). S. Lin et al. (2020) describe ceasing use, reducing use in terms of frequency and duration, or a person’s thoughts about changing their use in these respects as discontinuous intention to use. S. Kim et al. (2024) posits that when users experience social media fatigue, they perceive the time they have spent on the platform in question as not sufficiently beneficial, which in turn negatively influences their attitude toward the platform. This negative attitude then fosters the intention to discontinue use.

The many features and possibilities of social media lead people to spend more time using these platforms and to devote energy and attention to managing the content and interactions (Maier et al., 2012). Users with higher usage intensity are more likely to experience social media fatigue (Luqman et al., 2017; Malik et al., 2020). Users may also have difficulty coping with the flood of information and communication on social media. This condition is referred to as social media overload (Maier et al., 2015 cited in Whelan et al., 2020, p. 869; S. Zhang et al., 2016). When processing social media content becomes overwhelming for a person, this can increase the likelihood of experiencing fatigue (Whelan et al., 2020). The following sections address overload as a contributing factor to social media fatigue.

### 2.3. Overload from SNSs

Overload is defined as a person’s subjective perception and assessment of the amount of people, information, and objects that they can no longer process (Saegert, 1973). There are various types of overload considered in the context of SNSs, such as system feature overload, information overload, social overload, communication overload, and connection overload (Grandhi et al., 2005; S. Zhang et al., 2016; Maier et al., 2014; Lee et al., 2016; LaRose et al., 2014).

- System feature overload occurs when a social network is perceived as too complex for the specific task at hand, or when new features exhibit excessive complexity that is disproportionate to the increased technical effort or the heightened complexity of use (Karr-Wisniewski & Lu, 2010; Lee et al., 2016).
- Connection overload describes the strain and stress associated with updating, maintaining, and constantly receiving messages, which can accompany the use of SNSs (LaRose et al., 2014).
- When users feel they must provide too much social support to friends on SNSs, this can lead to social overload (Maier et al., 2014).

Although system feature, social, and connection overload are relevant forms of SNS-related strain, they may be less directly related to the specific research question examined here. System feature overload primarily concerns the complexity and usability of platform features, whereas the present study focuses on the demands arising from users’ exposure to content and communication while browsing LinkedIn. Social overload refers more specifically to the perceived obligation to provide social support to others, which may be particularly relevant in interpersonal or friendship-oriented SNS contexts. Connection overload, in contrast, concerns the burden associated with maintaining and receiving a large number of social connections and messages and therefore overlaps conceptually with communication-related demands. Given the professional and networking-oriented context of LinkedIn, information and communication overload were therefore selected as the two dimensions most directly relevant to the present research question. This selection does not imply that other forms of overload are irrelevant to LinkedIn use; rather, they were beyond the specific scope of the present study.

This study examines information overload and communication overload. These two types of overload are the focus of this research because, according to Cao and Sun (2017) and Lee et al. (2016), social media overload describes a state in which the intensive use of SNSs results in users being confronted with an extreme volume of communication requests and a high amount of information, leading to an excessive drain on users’ cognitive and energetic resources and exceeding their capacity to cope. Both types of overloads are considered stressors that can lead to negative consequences such as fatigue, discontinuation of network use, dissatisfaction, and emotional exhaustion (Maier et al., 2015; Ragu-Nathan et al., 2008; Tarafdar et al., 2011). For this reason, communication overload and information overload are two key aspects of social media overload.

The following sections explore these two aspects of overload in greater depth.

#### 2.3.1. Communication Overload and Social Media Fatigue

Communication overload refers to a person’s subjective perception that their own processing capacity is being exceeded by requests for conversation and communication via SNSs (Saegert, 1973; K. Li et al., 2024). Constant communication can lead to an imbalance between users’ need for communication and their cognitive abilities, resulting in their inability to cope with the amount of communication requests (Cao & Sun, 2017; K. Li et al., 2024). Like social media fatigue, communication overload is subjective; thus, the same level of communication may be difficult for one person to manage and lead to fatigue, while another person may have no difficulty managing it and therefore becomes fatigued more slowly (Ravindran et al., 2014).

The increased amount of information, along with connections to and communication with more people, can influence users’ behavior and attention (Saegert, 1973; K. Li et al., 2024). The disruption of attention in everyday life caused by frequent communication requests from SNSs can lead users to invest more energy in order to return to their original state after the interruption. This fosters the excessive consumption of cognitive energy, which in turn can lead to social media fatigue (Cao & Sun, 2017; K. Li et al., 2024).

#### 2.3.2. Information Overload and Social Media Fatigue

Information overload caused by SNSs occurs when the amount of information that needs to be processed exceeds one’s information-processing capacity (Eppler & Mengis, 2004; Whelan & Teigland, 2013). One model that can be used to explain information overload is the limited capacity model (S. Zhang et al., 2021; Lang, 2000) which posits that people have a limited capacity to process information. If this capacity is exceeded, it can lead to negative consequences, such as increased stress levels, reduced decision-making ability, and cognitive overload. Being exposed to a variety of constantly updated content is likely to be experienced as an information overload. Attempting to cope with this flood of information from SNSs is likely to deplete cognitive resources leading to fatigue and exhaustion (Pang & Ruan, 2023; Shahrzadi et al., 2024; Lang, 2000).

The limited capacity model is relevant to research on information overload, as SNSs today expose people to an unprecedented amount of information that often exceeds their processing capacity. Users’ attempts to navigate the never-ending flood of information is likely to overwhelm their cognitive resources, which can lead both to problems understanding the information and difficulties in processing it (Ayyagari et al., 2011; S. Zhang et al., 2021). The Limited Capacity Model of Motivated Mediated Message Processing (LC4MP) also posits that humans have limited cognitive resources and that these resources are allocated to three processes:

- Encoding; that is, the reception and processing of information from media messages.
- Storage; that is, the transfer of encoded information into memory.
- Retrieval; that is, the ability to retrieve the stored information when needed (Lang, 2000, 2009).

If online content contains a multitude of stimuli, this can lead to an overload of cognitive resources, potentially resulting in information being encoded more superficially, stored poorly, or recalled only unreliably (Lang, 2009). When processing messages from a network, resource allocation constantly adapts to the information load, changes in stimulus characteristics, motivational relevance, and the level of activation within the user’s motivational system, as well as their intentions and goals for using the network. Cognitive overload occurs when insufficient resources are available for one of the processes—namely, encoding, storage, or retrieval. Another relevant influencing factor is how many resources the message itself requires (Lang, 2009).

According to S. Kim et al. (2024), the nature of the overload may vary across different types of SNSs. A distinction is made between text-based and image-based SNSs. Platforms such as Threads and Twitter are considered text-based SNSs. They are characterized by the fact that communication and interaction take place primarily through text, even though multimedia is permitted (S. Davis, 2023; Pittman & Reich, 2016; D. H. Kim et al., 2017). Instagram and Pinterest, on the other hand, are considered image-based networks, as the focus is on videos and images (D. H. Kim et al., 2017). Users of text-based SNSs share opinions, news, and experiences and engage in conversations in the comments section (S. Kim et al., 2024). The large amount of text on text-based networks requires increased cognitive effort, which can have a positive effect on information overload and, consequently, potentially also on social media fatigue (S. Kim et al., 2024; Sheng et al., 2022).

As mentioned in the introduction, a key factor to consider in research on SNSs and their effects on mental health is the nature of usage itself (i.e., passive or active; cf., Frison & Eggermont, 2015; Aikins, 2021; Verduyn et al., 2015; Escobar-Viera et al., 2018). To date, passive use in particular has been associated with negative emotions (Burke et al., 2010; Deters & Mehl, 2012). Therefore, the following sections address the types of use and the relationships between passive use and the constructs discussed.

### 2.4. Passive Use of SNSs

In terms of usage patterns, a distinction is made between active and passive use. Passive use refers to viewing other users’ content without attempting to interact with them (Tosun, 2012; Chen et al., 2016). It includes viewing the feed, photos, and videos, as well as reading comments, but does not include commenting on or sharing content oneself (Frison & Eggermont, 2015; Erliksson et al., 2020). Active use, on the other hand, is characterized by posting, commenting, participating in groups, and sending messages (Aikins, 2021). The key difference between the two types of use is whether some form of communication takes place via the social network (Zhu & Bao, 2018).

Studies examining the potential effects of passive social media use have found that it can be associated with reduced subjective well-being and symptoms of social anxiety (Zhu & Bao, 2018). Furthermore, passive use can lead to envy and feelings of loneliness (Krasnova et al., 2013; Burke et al., 2010). In their study, Duvenage et al. (2019) showed that students had more difficulty recovering from negative emotions when they relied on passive digital behaviors to cope with daily stressors. Passive use is often associated with negative consequences for users’ mental health (Aalbers et al., 2019). One reason for this may be that passive use inhibits the user’s emotions internally (Verduyn et al., 2015). Active use, on the other hand, leads to emotions being expressed outwardly through interactions, which, according to emotion regulation theory, is central to strengthening existing relationships with friends and obtaining social support (Myruski et al., 2019; Farmer & Kashdan, 2012; Nolen-Hoeksema, 2012). Suppressing negative emotions, on the other hand, has been shown to be ineffective in reducing them, a finding that is also reflected in the study results discussed by Duvenage et al. (2019) including (Farmer & Kashdan, 2012; Nolen-Hoeksema, 2012; Rasmussen et al., 2019).

With respect to LinkedIn, current research focuses primarily on the potential career benefits that may result from it. It has been shown that passive LinkedIn use offers informational advantages, as it facilitates the identification of potentially relevant contacts and thus makes it easier to reach out to them in a targeted manner. This is referred to as *ambient awareness* and refers to the metacognitive knowledge of *who knows whom* and *who knows what* (Leonardi, 2015). Active use, on the other hand, offers more benefits in terms of career and job opportunities as well as one’s personal image, since *social capital*—that is, the resources derived from social relationships—can be leveraged to one’s advantage in one’s professional career (H. Davis et al., 2020). A study by Bontcheva et al. (2013) also showed that users who post more rarely perceive the amount of posts as excessive. However, there has been no in-depth examination of the relationship between passive LinkedIn use and its potential negative consequences.

Although LinkedIn is discussed as a platform in research on passive social network use and its outcomes, it has rarely been examined in isolation (Ravindran et al., 2014). Professional SNSs differ substantially from private SNSs in terms of communication goals, self-presentation, and networking behavior, making platform-specific operationalizations particularly important. One reason for this limited evidence may be the lack of platform-specific measurement instruments capable of capturing different forms of LinkedIn usage behavior. Existing measures of SNS use are often generalized across platforms and frequently do not differentiate sufficiently between passive and active forms of professional social networking behavior. To address this limitation, the present study introduces the LinkedIn Activity Questionnaire (LAQ), which was developed to assess LinkedIn-specific usage patterns with a particular focus on passive use. Importantly, the distinction between LinkedIn and other social networking platforms should not be understood as the absence of interpersonal or private communication affordances. LinkedIn also enables direct messaging, social interaction, profile viewing, and other forms of interpersonal exchange. Rather, these affordances are embedded within a predominantly professional and instrumental context, in which communication and networking may simultaneously serve career-related goals, professional self-presentation, and relationship maintenance. Thus, the relevant distinction may concern not the presence versus absence of particular affordances, but the functional context in which users encounter and respond to them.

### 2.5. The Relationship Between Passive Use, Overload, and Social Media Fatigue

Current research examining the link between social media fatigue and passive use focuses on social media fatigue and overload (Zhu & Bao, 2018; Bright et al., 2015). Ravindran et al. (2014), for example, argue that users change the nature of their usage to prevent social media fatigue. Consequently, when they feel overwhelmed, they limit their participation in the social network (Bright et al., 2015). Zhu and Bao (2018) also assumed that social media fatigue promotes passive use.

However, the distinction between active and passive social media use should not be regarded as a sufficient theoretical explanation of users’ well-being outcomes. Recent reviews have questioned the assumption that passive use is inherently detrimental and have shown that associations between active or passive use and well-being are generally small and dependent on contextual and methodological factors (Valkenburg et al., 2022; Godard & Holtzman, 2024). Rather than treating passive use as inherently harmful, it may therefore be more useful to examine the specific demands and experiences associated with forms of use. In the present study, passive use is consequently conceptualized as a behavioral starting point that may increase exposure to information- and communication-related demands, while overload represents the proximal psychological mechanism through which such exposure may be associated with social media fatigue.

From this perspective, the relationship between passive LinkedIn use and social media fatigue may be better understood as an indirect process rather than as a direct association. Passive use primarily describes a behavioral pattern characterized by non-interactive browsing and monitoring. Such use may increase exposure to information, messages, connection requests, and other communication-related demands characteristic of LinkedIn. However, exposure to these demands does not necessarily result in fatigue in itself. Rather, their psychological impact may depend on the extent to which users perceive the amount of information or communication to exceed their individual coping capacities.

This distinction is consistent with the Stressor–Strain–Outcome framework (Koeske & Koeske, 1993). Within the present study, communication overload and information overload can be conceptualized as proximal stressors, whereas social media fatigue represents the resulting strain. Passive LinkedIn use is therefore conceptualized as an antecedent that may increase exposure to potential stressors, rather than as the stressor itself. Accordingly, the theoretical rationale for the present mediation model is that passive use may contribute to overload, which in turn may increase social media fatigue. This framework suggests that passive LinkedIn use does not necessarily need to be directly associated with fatigue if its effects operate primarily through users’ subjective perceptions of communication and information overload.

Thus, although a direct association between passive LinkedIn use and social media fatigue was examined in H1, we expected the theoretically more proximal mechanisms of communication overload and information overload to account for the association between passive use and fatigue. This distinction also allows for the possibility that passive use may be experienced differently across individuals depending on their perceived capacity to process incoming information and communication.

However, the effect of passive use on social media fatigue has not yet been explored in detail, although some studies have examined the intention to use SNSs passively (Tian et al., 2024; J. Li et al., 2021), but not current usage behavior. The present study, on the other hand, examines individuals’ current usage behavior and aims to determine whether passive use can trigger social media fatigue, as passive use has been shown to have negative consequences for users’ mental well-being.

Importantly, the professional context of LinkedIn may alter the relative relevance of different forms of overload compared with private social networking sites. LinkedIn is primarily oriented toward professional networking, career-related information, self-presentation, and maintaining professional relationships, rather than primarily toward interpersonal entertainment or social interaction. Consequently, platform-related demands may not be limited to the amount of content encountered. Even users who primarily engage in passive browsing may receive connection requests, messages, notifications, and other communication-related demands from professional contacts. Thus, passive use on LinkedIn may be particularly relevant to communication overload because users can remain exposed to communication demands despite limiting their own active participation. In contrast, the lower intensity of content production and the comparatively lower frequency of LinkedIn use may reduce the extent to which passive browsing translates into information overload. This suggests that, in professional SNS contexts, communication overload may represent a more proximal mechanism linking passive use to social media fatigue than information overload.

To determine whether the reverse effect also exists (media fatigue causing using behavior) and to address the existing research gap regarding the use of professional SNSs and social media fatigue and overload, this study examines the following research question: Is passive LinkedIn use associated with social media fatigue through communication overload and information overload?

The research hypotheses are:

**H1.**  *Passive LinkedIn use is positively associated with social media fatigue.*

**H2a.**  *Passive LinkedIn use is positively associated with communication overload.*

**H2b.**  *Passive LinkedIn use is positively associated with information overload.*

**H3.**  *Communication overload is positively associated with social media fatigue.*

**H4.**  *Information overload is positively associated with social media fatigue.*

**H5.**  *The positive relationship between passive LinkedIn use (IV) and social media fatigue (DV) is mediated H5a by communication overload (M1a) and H5b by information overload (M1b).*

Although H1 tested the overall association between passive LinkedIn use and social media fatigue, our theoretical model assumed that this association would primarily emerge through the proximal mechanisms of communication overload and information overload.

## 3. Method

A non-experimental, quantitative, one-time online questionnaire was employed to investigate the research question and hypotheses. The questionnaire was created and administered using the EFS Survey (Tivian XI GmbH, Cologne, Germany; https://www.unipark.de/, accessed 18 August 2026). A pretest was conducted with *N* = 5 participants in order to identify ambiguities in content as well as technical errors. After the problems identified by the pretest were resolved, the official survey began on 28 May 2025 and ended on 3 July 2025. The average completion time of the questionnaire was 15.45 min. The survey was conducted in collaboration with another student, which is why the questionnaire collected data on constructs that are not considered in this report.

### 3.1. Participants

The questionnaire was shared via social media platforms such as WhatsApp, Instagram, and LinkedIn. In addition, friends and family members were personally asked to participate and invited to share the survey link. As an incentive for students to participate, they were compensated with subject hours. The sample is a convenience sample which included participants who could be reached at the time the survey was conducted.

To determine the required sample size, a prior power analysis was conducted using the G*Power program (version 3.1.9.7). Since, in addition to the three central variables, a control variable, the duration of use, was to be taken into account, a necessary sample size of *N* = 132 was calculated for a multiple regression with four predictors, an effect size of *f*^2^ = 0.15, a significance level of *α* = 0.05, and a power of 1 − *β* = 0.95.

To participate in the study, participants had to use LinkedIn and be of legal age. A total sample of 445 people was reached, meaning that 445 people opened the survey. However, only 31.46% completed the survey, resulting in a sample size of 140. However, three participants had to be excluded due to too many missing values, resulting in a final sample size of *N* = 137. Of the 137 individuals, 84 were female and 53 were male; no one identified as non-binary. The average age was 31.95 years with a standard deviation of SD = 10.04. The youngest participant was 21 years old and the oldest was 62 years old. Regarding the highest level of education, 75.18% of the participants reported holding an academic degree (Bachelor’s, Master’s, or Diploma), 24.09% reported a vocational diploma or high school diploma, and one person reported a secondary school diploma. Moreover, 68.61% of the sample consisted of employees, 25.55% were students, 2.19% reported being self-employed, and 3.65% of the sample were job seekers at the time of the survey (Table A9). Most respondents (61.31%) reported working full-time (35 or more hours per week), while 14.6% were working students and 13.14% worked part-time (20 to fewer than 35 h per week) (Table A11). The distribution of work experience presented a mixed picture. The majority of respondents had one to four years of work experience (32.1%), followed by those with five to ten years (17.5%) and 11–20 years (16.8%). Moreover, 13.1% of the participants reported having less than one year of professional experience, while 7.3% had no professional experience at all. Longer periods of employment were less common; 9.5% of the participants reported having 21–30 years of professional experience, 2.9% had 31–40 years, and only 0.7% had 41–50 years (see Appendix A).

### 3.2. Materials

Participants were informed that participation was voluntary and that their identities would remain anonymous. They received information regarding the General Data Protection Regulation.

Demographic data was collected. The survey asked about age, gender, highest level of education, current employment status, ability to work, work experience, whether the respondent held a leadership role, the frequency of LinkedIn use per week, and which notification settings were enabled on LinkedIn. Next, LinkedIn usage was assessed. In addition, information overload, communication overload and social media fatigue were measured and participants were asked regarding their intention to discontinue use of LinkedIn.

All questionnaires used were translated from English into German by the study investigators using the backtranslation method by Brislin (1970) adapted to the LinkedIn context. For example, the term “Friends” was replaced with “Contacts” in the adapted version, since LinkedIn is a professional rather than a personal social network. Similarly, “friend requests” were rephrased as “connection requests”.

To measure (passive) LinkedIn use, we developed the LinkedIn Activity Questionnaire (LAQ) (see Appendix B). We derived the new measure from the XING Activity Questionnaire (XAQ) by Brandenberg et al. (2019). The XAQ originally contained 27 items including the following six subscales: Watching Compare (α = 0.87), Watching Inform (α = 0.77), Acting Global (α = 0.83), Acting Dyadic (α = 0.73), Acting Career (α = 0.80), and Impressing (α = 0.74). The Watching Inform and Watching Compare scales represent passive use, while the other scales reflect active use. The 27 items exhibited an overall internal consistency of *α* = 0.92 in the previous study. In the present study, the XAQ items were adapted to the LinkedIn context to derive a preliminary LinkedIn-specific operationalization of usage behavior (LAQ). Thus, the LAQ should not be understood as an entirely novel measure developed de novo, but as an adaptation of an existing measure of professional social media use. Exploratory factor analysis suggested a three-factor structure of the LAQ including 24 items representing Passive Use, Active Use, and Career Management. Because passive use is the behavioral focus of the present study, subsequent analyses focused on this dimension. In the present study, passive LinkedIn use was conceptualized as non-interactive browsing and monitoring behavior rather than exclusively as the consumption of content produced by other users. Accordingly, the passive-use dimension includes behaviors such as viewing other users’ profiles and LinkedIn content, browsing company profiles, searching for known contacts, and monitoring one’s own profile-related activity. These behaviors do not involve actively producing content or directly communicating with other users but reflect observational or monitoring-oriented engagement with the platform. Some of these behaviors, particularly checking one’s own profile statistics, searching for known contacts, and viewing profile visitors, may also reflect instrumental career management or self-monitoring. Their assignment to the passive-use dimension was therefore based on the exploratory factor structure and the content similarity of the items rather than on the assumption that they represent passive content consumption *in a strict sense*.

Information overload was measured using the questionnaire developed by S. Zhang et al. (2016), which has a good Cronbach’s alpha value of *α* = 0.86. The questionnaire consists of four items, and responses are rated on a seven-point Likert scale (ranging from “strongly disagree” to “strongly agree”). An example item from the questionnaire is: I am often distracted by the excessive amount of information available to me on LinkedIn.

The questionnaire developed by Lee et al. (2016) was used to assess communication overload. The Cronbach’s alpha value for this questionnaire is *α* = 0.82, which is considered good. It contains five items on a seven-point Likert scale (from “strongly disagree” to “strongly agree”) and was measured, for example, by the following item: I receive more messages and updates from contacts on LinkedIn than I can handle.

The questionnaire developed by S. Zhang et al. (2021) was used to measure social media fatigue by 15 items, with five items each corresponding to the behavioral, emotional, and cognitive levels. One item on the behavioral level is: I find it difficult to come up with good ideas for posts on LinkedIn. An item measuring the emotional level is: I feel nervous when I receive connection requests on LinkedIn. And an item measuring the cognitive level is: I am often overwhelmed by the amount of information available on LinkedIn. Cronbach’s Alpha was calculated for internal consistency, yielding a value of *α* = 0.83. The subscale for cognitive fatigue yielded a value of *α* = 0.88, for behavioral fatigue *α* = 0.69, and for emotional fatigue *α* = 0.71. Responses were given on a seven-point Likert scale (strongly disagree to strongly agree). Values of 0.80 and above are considered as good, values of 0.70 or higher are considered as acceptable, and values of 0.60 or lower indicate limited reliability (McNeish, 2017; Hair, 2014).

Discontinuous usage intention was measured using the questionnaire developed by S. Kim et al. (2024), which consists of four items and is rated on a five-point Likert scale (ranging from “strongly disagree” to “strongly agree”). One item on this scale, for example, reads: If I could, I would stop using LinkedIn. The reliability and validity of the scale were calculated using composite reliability and average variance extracted, with values of CR = 0.875 and AVE = 0.64. The CR value indicates high internal consistency, and the AVE suggests good convergent validity (Fornell & Larcker, 1981; Hair, 2014).

### 3.3. Statistical Analysis

The statistical analysis was conducted using R Studio (version 4.4.2). To develop the LinkedIn Activity Questionnaire, items from the XAQ were adapted with respect to LinkedIn. Factor analysis was employed to derive independent subcales. Both analytical and interpretive objectivity are given (Moosbrugger & Kelava, 2020). Finally, for the constructs of communication overload, information overload, discontinuous usage intention and for the Social Media Fatigue Scale, mean scores were calculated for each participant across the respective items and a total score was computed.

To test hypotheses H1, H2a, H2b, H3, and H4, a Pearson product–moment correlation was calculated for metric variables (Rasch et al., 2014). Calculating these correlations allows for the examination of bivariate relationships between the relevant variables. The prerequisites were checked prior to the calculation. To this end, the variables were tested for normal distribution using skewness, kurtosis, and the Shapiro–Wilk test. The test was two-tailed at a significance level of *p* < 0.05. A correlation of 0.10 is interpreted as a weak effect, a value of 0.30 or higher is considered a moderate effect, and a correlation value of 0.50 or higher is classified as a strong effect (Bortz & Döring, 2013).

To test H5, a parallel mediation analysis was conducted. The PROCESS model by Hayes (Hayes, 2017) was used for this purpose. Passive LinkedIn use was the independent variable (IV), social media fatigue was the dependent variable (DV), communication overload represented mediator one (M1), and information overload represented mediator two (M2). In addition, frequency of use was included as a covariate, because it may influence the experience of social media fatigue, requiring control for this variable (Luqman et al., 2017; Malik et al., 2020). Bootstrapping was used to estimate the indirect effects, with 10,000 samples drawn. A bias-corrected 95% confidence interval was also used. An indirect effect is interpreted as significant if the confidence interval does not include the value zero.

At the correlational level, the relationship between social media fatigue, communication overload, information overload, passive LinkedIn use and frequency of use was examined in relation to discontinuous usage intention. Confirmation of statistical assumptions was first tested employing a significance level of *p* < 0.05.

Internal consistency was calculated for all scales and an exploratory factor analysis was also conducted. For the LinkedIn Activity Questionnaire, this was done to determine which items reflect passive usage behavior and which reflect active usage behavior. The exploratory analysis was performed using principal axis factor analysis with oblique rotation for the scales to examine their dimensional structure. The number of factors to be extracted was determined in each case using a parallel test. Prior to this, the suitability of the data for factor analysis was verified using the Kaiser–Meyer–Olkin (KMO) test and Bartlett’s test (Kaiser, 1974). The evaluation of Cronbach’s alpha was based on the thresholds established by George and Mallery (2016) as well as Moosbrugger and Kelava (2020), according to which values of 0.70 or higher are classified as acceptable and values of 0.80 or higher are considered as good.

## 4. Results

The findings of the exploratory factor analysis, the descriptive and inferential statistical analyses, and the results of the exploratory analysis are discussed and presented in the following sections.

### 4.1. Exploratory Factor Analysis of the Scales

The LinkedIn Activity Questionnaire (*KMO* = 0.82) and the Social Media Fatigue Scale (*KMO* = 0.84) yielded good values, the Communication Overload Scale (*KMO* = 0.76) yielded an acceptable value, and the Information Overload Scale yielded a nearly acceptable value (*KMO* = 0.68). All Bartlett’s tests were significant (*p* < 0.001), so the data can be considered suitable for factor analysis overall (see Appendix A).

For the LinkedIn Activity Questionnaire, the parallel test yielded a four-factor solution. Given the conceptual similarity of the items and the exploratory purpose of the analysis, these items were combined into a single passive-use factor. Items that loaded onto factors 1 and 3 were thus combined into a single factor based on content considerations (i.e., Factor 1 = Passive LinkedIn Use) resulting in a preliminary three-factor solution adopted for exploratory purposes. Passive use is represented by eleven items (BV_1, BV_2, BV_3, BV_4, BV_5, BV_6, BI_1, BI_2, II_3, SD_3, SD_4) (Table A13). The internal consistency of the scale is good, with a Cronbach’s alpha of *α* = 0.87 (Table A12). These findings provide initial support for the internal consistency and structural differentiation of the passive-use dimension of the LAQ (see Appendix A).

An exploratory factor analysis was also conducted for the Social Media Fatigue Scale (Table A9). As in the original version, this yielded three factors. Factor one included all items from the cognitive level as well as one item from the emotional level. However, not all items loaded onto factors two and three as they did in the original version. Based on the content of the items, it became apparent that all items in factor two were related to forgetting something or addressed negative emotions toward LinkedIn. In factor three, only two items loaded well, both of which addressed not knowing what to post. However, to assess how well the items represented the originally intended dimensions, Cronbach’s alpha was calculated for the original structure. The cognitive scale exhibited good internal consistency (*α* = 0.88), while the behavioral scale showed only moderate and questionable consistency (*α* = 0.69), whereas the emotional scale had acceptable internal consistency (*α* = 0.72). The Social Media Fatigue Scale showed good overall internal consistency with *α* = 0.87 (Table A12). The internal consistency of the Communication Overload Scale was *α* = 0.83 (Table A12), which is considered good; the parallel analysis suggested a two-factor solution (Table A10). The Information Overload Scale showed acceptable internal consistency of *α* = 0.77 (Table A12). The scale for measuring discontinuous usage intention had acceptable internal consistency of *α* = 0.73 (Table A12). The Information Overload Scale proved to be unidimensional (Table A11).

### 4.2. Descriptive Statistics

Following the exploratory factor analyses, descriptive statistics were calculated. First, the weekly frequency of use yielded a median of *Md* = 2.0, which corresponds to one to two hours of use. However, 38.7% of the participants reported using LinkedIn for less than one hour per week, 31.4% of the participants used LinkedIn for one to two hours per week, while 23.4% spent three to four hours per week on LinkedIn. Only 5.8% reported spending five to six hours per week online on LinkedIn, and only one person used LinkedIn for seven to nine hours per week (Table A1). To assess passive usage, a mean score of the passive items was calculated for each person. This resulted in a mean of *M* = 2.79 (*SD* = 0.66). This procedure was applied to all scales. The mean score for communication overload was *M* = 2.89 (*SD* = 1.3), and for information overload it was *M* = 3.88 (*SD* = 1.21). For social media fatigue, a mean of *M* = 3.04 (*SD* = 0.98) was calculated across all items. The mean for discontinuous usage intention was M = 2.45 (*SD* = 0.84) (Table A6).

Table 1 summarizes the means and standard deviations for each subscale.

### 4.3. Hypothesis Testing

First, the preconditions for performing a parallel mediation analysis for all key variables were examined.

#### 4.3.1. Correlation Analyses

Bivariate Pearson correlations were calculated between the key variables. The analyses revealed that passive LinkedIn use was not significantly correlated with either information overload (*r*(137) = 0.015, *p* = 0.859) or social media fatigue (*r*(137) = 0.017, *p* = 0.841). Hypotheses H1 and H2b could therefore not be confirmed. A significant, albeit only weakly positive, correlation was found between passive use and communication overload *r*(137) = 0.183, *p* = 0.033. Hypothesis H2a was therefore confirmed. There was a moderate and significant correlation between communication overload and social media fatigue (*r*(137) = 0.524, *p* < 0.001), which confirms Hypothesis H3. Similarly, a strong positive correlation was found between information overload and social media fatigue, *r*(137) = 0.646, *p* < 0.001, thus confirming Hypothesis H4. To account for the potential influence of the control variable (weekly usage frequency), partial correlations were also calculated. Even when controlling for this variable, the described relationships remained intact. Only a slight increase in the correlation coefficient between passive LinkedIn use and social media fatigue emerged; however, the relationship did not remain significant. The correlation between passive LinkedIn use and communication overload also increased slightly. The other correlation coefficients remained virtually unchanged. Thus, hypotheses H2a, H3, and H4 were confirmed, while H1 and H2b were refuted (Table A12 and Table A13).

#### 4.3.2. Parallel Mediation Analysis

The mediation model focused on social media fatigue as dependent variable. The parallel mediation analysis, with passive LinkedIn use as the predictor, social media fatigue as the criterion variable, and communication overload and information overload as parallel mediators, yielded a significant overall model, *F* (3;133) = 39.551, *p* < 0.001, with an explained variance of *R*^2^ = 0.472. Thus, the model explains remarkable 47.2% of the variance of Social Media Fatigue.

How is social media fatigue determined? The total effect of passive LinkedIn use on social media fatigue was not significant, *β* = 0.017, 95% *CI* [−0.228, 0.279], *p* = 0.841. The direct effect of passive LinkedIn use on social media fatigue was also not significant, *β* = −0.041, BC 95% *CI* [−0.248, 0.135], *p* = 0.528. The indirect effect via both mediators was also not statistically significant, because the confidence interval included the value zero, *β* = 0.058, BC 95% *CI* [−0.072, 0.188].

However, passive LinkedIn use was in correspondence with H5a a significant predictor of communication overload, *β* = 0.183, 95% *CI* [0.019, 0.676], *p* = 0.033, whereas, in contrast to H5b, no significant association was found with information overload, *β* = 0.015, 95% *CI* [−0.308, 0.347], *p* = 0.859. Communication overload proved to be a significant predictor of social media fatigue (*β* = 0.275, 95% *CI* [0.082, 0.336], *p* < 0.001), as did information overload (*β* = 0.507, BC 95% *CI* [0.292, 0.532], *p* < 0.001). Finally, an indirect effect via communication overload was obtained in correspondence with H5a (*β* = 0.050, BC 95% *CI* [0.002, 0.117]), but not via information overload (in contrast to H5b; *β* = 0.008, 95% *CI* [−0.085, 0.100]). Therefore, only communication overload (but not information overload) mediated the influence of passive media use on social media fatigue.

**Figure 1:**
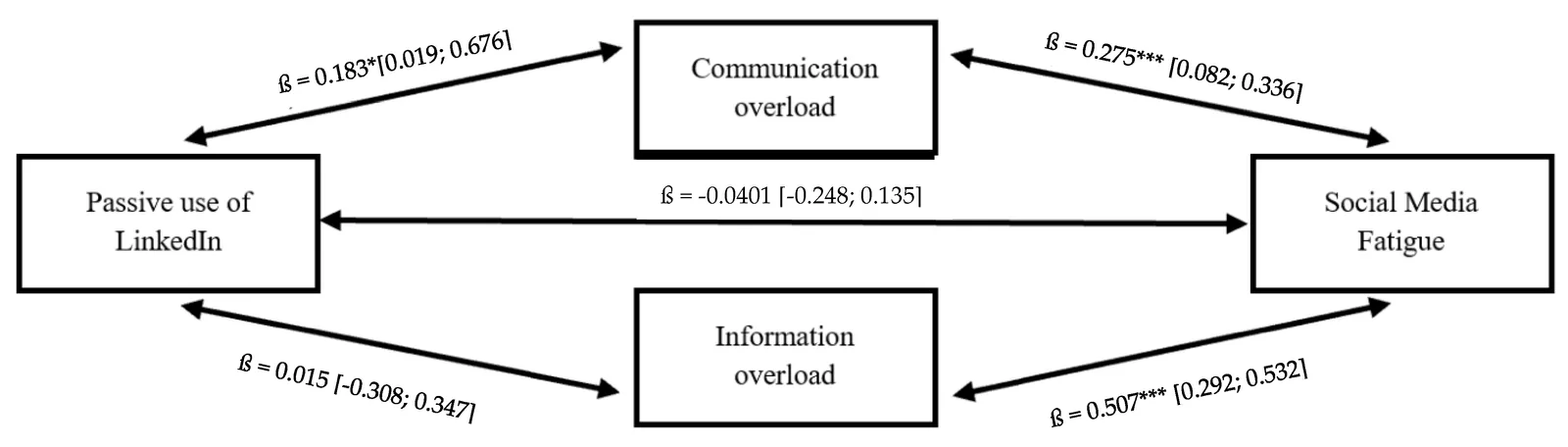
Results of the hypothesis tests H5. *Note*. m = 10,000; bootstrap intervals in square brackets; frequency of use (*β* = 0.117, 95% BC *CI* [−0.014; 0.247]) as a covariate showed no significant effect on the statistical mediation model. *p* < 0.05 *, *p* < 0.001 ***.

Thus, the specific indirect effect through communication overload was significant, whereas the specific indirect effect through information overload was not, supporting H5a but not H5b. Even after including the covariate “frequency of LinkedIn use per week,” the model fit and the direct effects of communication overload and information overload on social media fatigue remained virtually unchanged. The total indirect effect across both mediators increased slightly; however, the confidence interval continued to include the value zero and thus remained statistically insignificant. The H5 hypothesis tests are summarized in Figure 1.

#### 4.3.3. Exploratory Analysis

The correlation between social media fatigue and the intention to discontinue use was significant and moderately positive, *r*(137) = 0.564, *p* < 0.001; the relationship remained significant and moderately positive even when controlling for duration of use, *r*(137) = 0.555, *p* < 0.001. Communication overload also showed a significant positive correlation with discontinuous usage intention *r*(137) = 0.288, *p* < 0.001, as did information overload *r*(137) = 0.390, *p* < 0.001. Passive LinkedIn use revealed a weak negative and statistically insignificant correlation with discontinuous usage intention *r*(137) = −0.114, *p* > 0.05; the same applies to weekly usage frequency *r*(137) = −0.156, *p* > 0.05 (Table A13).

## 5. Discussion

### 5.1. Summary

Hypotheses H1 and H2b could not be confirmed, because the bivariate correlations showed that passive LinkedIn use was not significantly associated with either information overload or social media fatigue. Hypothesis H2a, on the other hand, was confirmed, because a significant association between passive LinkedIn use and communication overload was observed. In addition, a moderate to strong association between both overload dimensions and social media fatigue was obtained, confirming H3 and H4. Therefore, both types of overload played a central role in the experience of social media fatigue.

Even after controlling for frequency of use, the key associations remained unchanged. It is particularly noteworthy that the association between passive LinkedIn use and information overload increased slightly when controlling for the covariate. The parallel mediation analysis provided further insights. The overall model was significant, but the total and indirect effects were not. The findings indicate a significant indirect pathway via communication overload, while the overall indirect effect remained non-significant. The research question of this study was therefore not unequivocally confirmed. The exploratory analyses revealed a moderate and stable association between social media fatigue and the intention to discontinue use, suggesting potential behavioral consequences of social media fatigue. Users who experience greater fatigue from SNSs thus have a heightened intention to reduce or discontinue their use. The overload dimensions also showed a correlation with the intention to discontinue use; however, passive LinkedIn use and frequency of use showed no correlation with the intention to discontinue use.

### 5.2. Interpretation and Theoretical Implications

The present study makes two primary contributions. First, it examined whether passive LinkedIn use is associated with social media fatigue through communication and information overload. Second, it introduced the LinkedIn Activity Questionnaire (LAQ) as a platform-specific measure of LinkedIn usage behavior. The results provide initial support for the usefulness of the LAQ in assessing passive LinkedIn use, while its psychometric properties require further validation in independent samples. Regarding the substantive findings, passive LinkedIn use was not directly associated with social media fatigue. Theoretically, the findings contribute to existing models of social media fatigue by suggesting that the relative importance of different overload mechanisms may depend on the context and functional characteristics of the platform. In the present study, communication overload, rather than information overload, emerged as the specific pathway linking passive LinkedIn use to social media fatigue. Given LinkedIn’s professional and networking-oriented context, this pattern suggests that passive use may be particularly relevant when users remain exposed to communication-related demands despite limited active participation. However, because the present study did not directly compare LinkedIn with private social networking platforms, this platform-specific interpretation should be considered a theoretical proposition that requires direct cross-platform testing.

In general, empirical evidence is available both for and against the notion that passive usage behavior negatively influences well-being. A study by X. Zhang et al. (2020) showed that both types of use might foster the experience of negative emotions. Note that active and passive use tend to be strongly associated with one another (Meier & Krause, 2022). It could therefore also happen that active use can lead to negative consequences such as social media fatigue.

The lack of a significant association between passive LinkedIn use and information overload might be due to the fact that LinkedIn is generally used less frequently (Bontcheva et al., 2013). This conclusion corresponds with the fact that users post on LinkedIn less than on private SNSs. A study by Bontcheva et al. (2013) found that 73.4% of users of professional SNSs were able to manage their feed, and only 26.6% reported occasionally missing posts or losing track of them. This study also found no significant correlation between reading posts and information overload. The participants reported feeling less overwhelmed by professional SNSs than by private SNSs, although they did not necessarily cope better with the number of posts. The usage frequency also reflects this infrequent use of the platform in our study, as the majority of users spend less than one hour per week or just one to two hours per week on LinkedIn.

Furthermore, fewer posts are formulated on professional platforms. In the study by Bontcheva et al. (2013), 88.3% of participants reported posting monthly or less frequently, while 70.3% posted at least weekly on private SNSs. Based on this, it can be assumed that the lower usage intensity and reduced number of posts compared to private SNSs resulted in a lower likelihood of information overload. In the present study, after controlling for covariates, it was found that higher usage frequency was associated with less communication and information overload, which could be attributed to the fact that LinkedIn users who use the network more regularly stay better informed and are thus not confronted with too much new information and communication requests when using it (Bontcheva et al., 2013).

In contrast, passive use of LinkedIn was significantly associated with communication overload, whereas no significant association was found with information overload. Although the association between passive LinkedIn use and communication overload was statistically significant, its magnitude was relatively small (r = 0.183). Therefore, the practical significance of this relationship should be interpreted cautiously. Given the relatively low and restricted level of LinkedIn use in our sample, the observed association may not generalize to users with substantially higher levels of platform engagement. This distinction may reflect the specific characteristics of LinkedIn as a professional social networking site. Unlike many private social networking platforms, LinkedIn is characterized not only by content consumption but also by professional networking, connection requests, messages, and other communication-related interactions. Passive users may therefore be exposed to communication-related demands even when they do not actively interact with other users themselves.

The non-significant association between passive use and information overload should not be interpreted as evidence that information overload is irrelevant to social media fatigue. Indeed, information overload was significantly associated with social media fatigue in the present study. Rather, the findings suggest that information overload may represent a more general correlate of fatigue, whereas communication overload may constitute a more proximal mechanism through which passive LinkedIn use is associated with fatigue. This interpretation is consistent with the specific indirect effect observed for communication overload, whereas no indirect effect was found through information overload. Further research is needed to determine whether this distinction is specific to professional SNSs or reflects differences in how individuals perceive and respond to communication and information demands.

In line with previous research, both communication overload and information overload were significantly associated with social media fatigue (S. Kim et al., 2024; Adhikari & Panda, 2019; Baj-Rogowska, 2023). However, the present findings indicate that these dimensions may play different roles in the relationship between passive LinkedIn use and fatigue. Whereas both forms of overload were related to fatigue, only communication overload constituted a significant pathway linking passive LinkedIn use to fatigue. The pattern is compatible with an inconsistent mediation structure because the estimated indirect effect through communication overload was positive, whereas the estimated direct effect was negative. However, because the direct effect was not statistically significant, this pattern should not be interpreted as evidence for a robust competing direct pathway.

The absence of significant direct associations is also noteworthy in light of the relatively low frequency of LinkedIn use in our sample. More than 60% of participants reported using LinkedIn for no more than two hours per week. It is therefore possible that the relatively limited exposure to the platform attenuated the associations between passive use, information overload, and social media fatigue. The observed null findings may thus reflect, at least in part, the relatively low intensity of LinkedIn use in the present sample. However, a specific threshold of use cannot be inferred from the present data and should be examined in future research with samples showing greater variability in LinkedIn use.

These findings are consistent with the Stressor–Strain–Outcome framework, in which overload represents a stressor and social media fatigue represents a resulting strain. The present findings extend this perspective by suggesting that, in the context of passive LinkedIn use, communication overload may represent a particularly relevant proximal stressor. From a practical perspective, reducing unnecessary communication demands, such as excessive notifications or unsolicited communication requests, may therefore help to reduce users’ experience of overload. However, such implications should be interpreted cautiously given the cross-sectional design of the present study. At the same time, communication overload should not be understood solely as a matter of individual self-regulation. Platform design may shape the amount and frequency of communication demands to which users are exposed. On LinkedIn, features such as notifications, connection requests, profile-related updates, and prompts to engage with other users may generate recurring communication demands even when users primarily browse the platform passively. From this perspective, communication overload may partly reflect the interaction between users’ coping resources and the communication environment created by the platform. Although the present study did not directly examine specific platform features or algorithmic processes, future research should investigate whether particular design characteristics contribute disproportionately to communication overload and whether design interventions could reduce unnecessary communication demands.

In this context, the *Theory of Planned Behavior* offers another explanatory framework (Ajzen, 1991). This theory assumes that individuals who perceive their behavioral control over an action as high are more likely to perform that action, even when faced with challenges related to it. Accordingly, reducing stressors associated with SNSs is likely to improve perceived behavioral control. Consciously managing one’s engagement with a social network is likely to lead to a reduction in overload and fatigue. Thus, greater control might influence the decision of whether or not to continue using a social network.

In summary, the results of this study suggest that, with regard to passive LinkedIn use, it is not so much the high amount of information (=information overload) but rather the communication overload that serves as a mediating factor in the experience of social media fatigue.

### 5.3. Limitations and Outlook

The present study does not allow causal conclusions because of its cross-sectional design (Setia, 2016). Although the mediation model was theoretically specified as passive LinkedIn use leading to communication overload and subsequently to social media fatigue, all variables were assessed at a single point in time. Therefore, the temporal ordering of these relationships cannot be established, and reverse or reciprocal relationships cannot be ruled out. For example, individuals experiencing greater social media fatigue may alter their LinkedIn usage behavior and engage more passively with the platform. Longitudinal or experimental studies are therefore needed to examine the temporal sequence of passive use, overload, and social media fatigue. Furthermore, the present study did not assess participants’ general use of private social media platforms. General social media use may represent an unmeasured confounding factor, as individuals who frequently use multiple social networking platforms may differ in their overall exposure to information and communication demands. Although the items assessing LinkedIn use were explicitly formulated to refer to LinkedIn, the potential influence of participants’ broader social media usage cannot be ruled out. Future studies should therefore assess general social media use and examine whether the observed associations remain when broader social media exposure is taken into account. Finally, the convenience-based recruitment procedure may have introduced self-selection bias. Participants were recruited through social media platforms, personal contacts, and subsequent sharing of the survey link, which may have resulted in the overrepresentation of individuals with a particular interest in LinkedIn or social media. This may limit the generalizability of the findings and potentially influence the observed associations. Future research should therefore aim to recruit more heterogeneous and representative samples.

The sample investigated in this study was relatively homogeneous in terms of LinkedIn usage frequency. Specifically, 38.7% of participants reported using LinkedIn for less than one hour per week and an additional 31.4% reported one to two hours per week, meaning that 70.1% of the sample used LinkedIn for no more than two hours per week. This restricted distribution may have limited the variability in LinkedIn exposure and potentially reduced the ability to detect associations between passive LinkedIn use and the study outcomes. In particular, the LinkedIn Activity Questionnaire (LAQ) should be considered a preliminary measure at this stage. Although the passive-use dimension showed good internal consistency, its dimensionality requires further validation. The exploratory analysis initially suggested a more differentiated factor structure, and two conceptually related factors were subsequently combined to operationalize passive LinkedIn use. This decision was based on exploratory factor-analytic results and content considerations and therefore requires independent confirmation. Moreover, as discussed above, some of the retained items may capture instrumental or self-monitoring behaviors in addition to passive browsing, further emphasizing the preliminary nature of the present operationalization. The present study does not provide independent cross-validation of this structure. Future studies should therefore examine the LAQ using confirmatory factor analysis in independent and larger samples and investigate whether the proposed passive-use dimension can be replicated or whether a more differentiated structure provides a better representation of LinkedIn usage behavior.

The cognitive subscale of the Social Media Fatigue Scale showed limited internal consistency in the present study (ω = 0.61), whereas the behavioral (ω = 0.76) and emotional (ω = 0.71) subscales showed acceptable reliability. This finding introduces some uncertainty regarding the precision with which cognitive fatigue was measured and therefore warrants caution when interpreting findings involving this subscale. Although the present study cannot determine the specific reasons for the lower reliability, differences in item interpretation, translation, or the breadth of the construct may be possible explanations. Future research should therefore further examine the cognitive fatigue items, including their linguistic and cultural appropriateness, and consider confirmatory validation and pilot testing in German-speaking samples.

The overload and social media fatigue measures were originally developed in cultural contexts different from the German context and were adapted using translation and backtranslation procedures. Although these procedures were intended to ensure conceptual and linguistic comparability, measurement invariance across cultural contexts was not assessed in the present study. Consequently, cross-cultural differences in the interpretation or functioning of individual items cannot be ruled out. Future research should therefore examine the measurement invariance of these instruments across relevant cultural and linguistic groups before making direct cross-cultural comparisons.

The questionnaires used for the measurement of the two overload dimensions were developed in Asia. Consequently, they were not formulated according to German standards, which may have had a negative impact, as some items consist of long sentences and contain various adverbs. Therefore, the items were not always formulated as concisely and precisely as possible (Benlidayi & Gupta, 2024). Furthermore, cultural differences were not considered, which is problematic, for example, when translating idioms and colloquial expressions (Beaton et al., 2000). Furthermore, a common-method bias may exist, as both the predictors and the criterion were collected using the same questionnaire (Rodríguez-Ardura & Meseguer-Artola, 2019).

Note that the statistical analysis did not distinguish between whether individuals used the platform purely passively or whether they used LinkedIn both actively and passively. For this reason, and due to the aforementioned divergences in the research regarding the active/passive dichotomy, future research should examine whether active use or the use of LinkedIn, in general, has an influence on the experience of social media fatigue (Meier & Krause, 2022). Furthermore, future research could determine whether the research question of this study applies to users who use LinkedIn exclusively in a passive manner.

When examining the outliers, it was noted that there were individuals whose overload scores were higher than their levels of social media fatigue. One reason for this could be that the individuals who perceive this overload are already taking steps to counteract social media fatigue through their behavior. Therefore, qualitative research would be of interest for follow-up studies.

Longitudinal studies would be a promising methodological approach in future research (Maxwell & Cole, 2007). Follow-up studies could also examine the individual dimensions of social media fatigue more closely. The present study indicated that the emotional subscale elicited on average the lowest scores. Follow-up studies could build on this finding.

Additionally, the LAQ is currently regarded as a preliminary measurement device. Although its passive-use dimension demonstrated good internal consistency, further validation using confirmatory factor analyses and larger samples is desirable.

### 5.4. Theoretical and Practical Implications

Beyond the theoretical contributions, the present study provides an initial LinkedIn-specific adaptation of an existing measure of professional social media use. Thus, the methodological contribution of the LAQ lies not in the development of an entirely novel measurement approach, but in adapting and preliminarily examining an existing behavioral measure for the LinkedIn context. Given its exploratory factor structure, however, the LAQ should be regarded as a preliminary operationalization that requires further psychometric validation.

At the theoretical level, the findings of this study contribute to the further development of existing models, as communication overload serves as a key mediator between passive LinkedIn use and social media fatigue. Information overload is not a mediator, but it is also significantly associated with social media fatigue. The results suggest that communication overload may represent a particularly relevant pathway linking passive use and social media fatigue in professional SNS contexts. The results are explained by models such as the Stress–Strain-Outcome model or the Limited Capacity model and are integrated within convincing theoretical approaches in this field of research (Sweller, 1988; Koeske & Koeske, 1993).

In practice, social media fatigue is interpreted as a sign that an individual’s threshold for social media stress has been exceeded, which can lead to withdrawal from the platform or taking more frequent breaks (Maier et al., 2015). In an organizational context and for HR managers, the results of this research offer opportunities to promote healthy user behavior among employees, for example, through training on self-regulation, notification management, and stress management, thereby counteracting fatigue in the long term (Peper & Harvey, 2018; Supriyadi et al., 2025). Individuals who feel fatigued by digital technologies should set limits for themselves and recognize that disconnecting from digital technologies, as well as taking breaks and practicing self-regulation, represent important interventions (Peper & Harvey, 2018). The present research revealed a mediating relationship between passive LinkedIn use, communication overload, and social media fatigue. Affected individuals should learn strategies to counteract this communication overload. To do so, users are advised to pay attention to their personal needs, for example, by disabling notifications or taking scheduled breaks to counteract the perceived overload (Reinecke & Oliver, 2016).

The importance of this topic in the workplace is supported by a study by van Zoonen and Rice (2017), which highlighted that while the use of social media for work-related purposes may be associated with greater autonomy, it is also linked to increased work pressure. Furthermore, a connection was revealed between social media use, autonomy, and exhaustion. The use of social media for work tends to intensify the work experience, which in turn is likely to be associated with increases in stress caused by interruptions or unpredictability (van Zoonen & Rice, 2017; Chesley, 2014).

A key influencing factor seems to be individuals’ responsiveness. Lower responsiveness has a positive impact on autonomy and exaggerated work engagement; however, in the long term, it does not necessarily lead to a reduced workload due to the constant flow of information and communication requests (van Zoonen & Rice, 2017).

According to Gibbs et al. (2013), a high amount of information and frequent communication due to the amount of content to be processed leads to a forced detachment from work. This underscores the urgency for organizations and users themselves to take action to address this issue. From the perspective of the LinkedIn platform itself, countering fatigue constitutes a strategic challenge, as a link between social media fatigue and discontinuous usage intent has been demonstrated. Therefore, the platform might introduce features that give users more control over the frequency of interactions and communications (Ruiz et al., 2024).

At the organizational and platform level, the findings also suggest that reducing unnecessary communication demands may require attention to the design and configuration of platform-based notifications and interaction prompts, rather than relying exclusively on individual self-regulation.

In conclusion, this study has provided new insights into the relationship between passive use of professional SNSs and the experience of social media fatigue, as well as the mechanisms underlying this relationship. Our findings suggest that the association between passive LinkedIn use and social media fatigue may be linked to communication overload.

In general, the findings of correlation analyses and mediation analysis have yielded both theoretical and practical implications that can lead to improvements in the practical use of professional SNSs. In addition, this study employed a promising new measurement device regarding the assessment of LinkedIn-specific usage behavior by the LinkedIn Activity Questionnaire.

## Figures and Tables

**Table 1:** Descriptive Statistics for the Social Media Fatigue Subscales.

Subscale	*M*	*SD*	Median	Skew	Kurtosis
Cognitive	3.27	1.41	3.20	0.20	−0.80
Behavioral	3.26	1.16	3.60	−0.28	−0.25
Emotional	2.57	1.06	2.60	0.40	−0.60

Notes. *M* = mean; *SD* = standard deviation; skewness = measure of the asymmetry of the distribution; kurtosis = measure of the peakedness of the distribution.

## Data Availability

The dataset generated and analyzed during the current study is available in the OSF repository: https://osf.io/h4v2z/files/osfstorage/6a8d4dbfb4f0221fede3a1c1, accessed on 18 August 2026.

## Appendix A. Supplementary Statistics

The following tables present the descriptive statistics for the calculations performed in the study.

**Table A1:** Frequency of LinkedIn use per week.

	*N*	%
Less than 1 h	53	38.7%
1–2 h	43	31.4%
3–4 h	32	23.4%
5–6 h	8	5.8%
7–9 h	1	0.7%

*Note*: *N* = 137.

**Table A2:** Descriptive statistics on the highest level of education attained.

	*N*	%
Junior High School Diploma	1	0.73%
Vocational Diploma/High School Diploma	33	24.09%
Academic degree (Bachelor’s, Master’s, Diploma)	103	75.18%

*Note*: *N* = 137.

**Table A3:** Descriptive statistics on Employment Status.

	*N*	%
Student	35	25.55%
Employee	94	68.61%
Self-employed	3	2.19%
Job seeker	5	3.65%

*Note*: *N* = 137.

**Table A4:** Descriptive statistics on Work Experience.

	*N*	%
No work experience	10	7.30%
Less than a year	18	13.14%
1–4 years	44	32.12%
5–10 years	24	17.52%
11–20 years	23	16.79%
21–30 years	13	9.49%
31–40 years	4	2.92%
41–50 years	1	0.73%

*Note*: *N* = 137.

**Table A5:** Descriptive statistics on Work Capacity.

	*N*	%
Unable to Work	8	5.84%
Part-time employment (mini-job)	7	5.11%
Working student	20	14.60%
Part-time (20 to less than 36 h per week)	18	13.14%
Full-time (35+ h per week)	84	61.32%

*Note*: *N* = 137.

**Table A6:** Descriptive statistics for main scales.

	*N*	Min.	Max.	M	SD
Passive use	137	1.18	4.55	2.79	0.66
Social Media Fatigue	137	1	5.78	3.04	0.98
Communication overload	137	1	6.6	2.89	1.3
Information overload	137	1.5	7	3.88	1.21
Intended use	137	1	5	2.45	0.84

*Note*: *N* = 137.

**Table A7:** Descriptive statistics for additional scales.

	*KMO*	*χ* ^2^	df	*p*
LAQ	0.82	1359.0	276	<0.001
Social Media Fatigue	0.84	851.58	105	<0.001
Communication overload	0.76	274.75	10	<0.001
Information overload	0.68	214.25	6	<0.001

*Note*: *KMO* = Kaiser–Meyer–Olkin test, *χ*^2^ = Bartlett test for sphericity.

**Table A8:** Exploratory factor analysis of the LinkedIn Activity Questionnaires: Structure matrix.

Items	Factor 1	Factor 2	Factor 3	Factor 4
AG_1		0.741		0.214
AG_2		0.660		
AG_3		0.643		
AG_4	0.228	0.610		−0.272
AG_5	0.224	0.206		
BV_1	0.437			0.500
BV_2	0.593			0.233
BV_3	0.673			
BV_4				0.726
BV_5	0.276			0.524
BV_6	0.536	0.249		
II_1		0.347		0.252
II_2		0.588		
II_3	0.708			
II_4	0.205	0.505		
BI_1	0.538			
BI_2		0.322	0.208	0.305
KA_1			0.621	
KA_2			0.823	
KA_3			0.788	
SD_1	0.507		0.358	
SD_2	0.322		0.240	
SD_3	0.577			
SD_4	0.349	0.230		0.240

*Note*: The extraction method is principal component analysis; the rotation method is Oblimin with Kaiser normalization.

**Table A9:** Exploratory Factor Analysis of the Social Media Fatigue Questionnaire: Structure matrix.

Items	Factor 1	Factor 2	Factor 3
SMF_1	0.864		
SMF_2	0.838		
SMF_3	0.824		
SMF_4	0.760		
SMF_5	0.554		
SMF_6			0.979
SMF_7		0.441	
SMF_8		0.476	0.232
SMF_9		0.624	
SMF_10			0.579
SMF_11		0.294	
SMF_12	0.432		
SMF_13		0.665	
SMF_14		0.769	
SMF_15		0.584	

*Note*: The extraction method is principal component analysis; the rotation method is Oblimin with Kaiser normalization.

**Table A10:** Exploratory Factor Analysis of the Communication Overload Scale: Structure matrix.

Items	Faktor 1	Faktor 2
CO_1		0.735
CO_2		0.829
CO_3	0.581	
CO_4	0.993	
CO_5	0.706	

*Note*: The extraction method is principal component analysis; the rotation method is Oblimin with Kaiser normalization.

**Table A11:** Exploratory Factor Analysis of the Information Overload Questionnaire: Structure matrix.

Items	Factor 1
IO_1	0.705
IO_2	0.957
IO_3	0.794
IO_4	0.263

*Note*: The extraction method is principal component analysis; the rotation method is Oblimin with Kaiser normalization.

**Table A12:** Correlation matrix.

Variable	Passive Use	SMF	CO	IO	NH	DNA
Passive use	1.00					
SMF	0.017	1.00				
CO	0.183 *	0.524 ***	1.00			
IO	0.015	0.646 ***	0.505 ***	1.00		
NH	0.416 ***	−0.141	−0.113	−0.129	1.00	
DNA	−0.114	0.564 ***	0.288 ***	0.390 ***	−0.156	1.00

*Note*: *N* = 137. p<0.05= *, p<0.001 = ***, SMF = Social Media Fatigue, CO = Communication overload, IO = Information overload, NH = Frequency of use, DNA = Discontinuous intention to use.

**Table A13:** Partial correlation matrix, taking usage frequency into account as a covariate.

Variable	Passive Use	SMF	CO	IO	DNA
Passive use	1.00				
SMF	0.084	1.00			
CO	0.254 **	0.517 ***	1.00		
IO	0.077	0.639 ***	0.510 ***	1.00	
DNA	−0.0416	0.555 ***	0.276 ***	0.378 ***	1.00

*Note*: *N* = 137. p<0.01 = **, p<0.001 = ***, SMF = Social Media Fatigue, CO = Communication overload, IO = Information overload, NH = Frequency of use, DNA = Discontinuous intention to use.

## Appendix B. LinkedIn Activity Questionnaire

**Table A14:** LinkedIn Activity Questionnaire.

Item	German	English
AG_1	Ich nehme an Diskussionen über Beiträge im Nachrichtenbereich teil.	I participate in discussions about posts in the news feed.
AG_2	Ich erstelle oder kommentiere Beiträge in Gruppen oder Foren.	I create or comment on posts in groups or forums.
AG_3	Ich teile Neuigkeiten.	I share news updates.
AG_4	Ich kommentiere Status-Updates anderer Nutzer:innen.	I comment on other users’ status updates.
AG_5	Ich überprüfe, ob meine privaten Nachrichten gelesen wurden oder nicht.	I check whether my private messages have been read or not.
BV_1	Ich sehe mir die Kontakte und Netzwerke anderer Nutzer:innen an.	I look at other users’ contacts and networks.
BV_2	Ich sehe mir die Profildetails anderer Nutzer:innen an (z. B. Berufserfahrung, Ausbildung, Organisationen, Interessen, persönliche Informationen).	I look at other users’ profile details (e.g., work experience, education, organizations, interests, personal information).
BV_3	Ich schaue mir die (neuesten) Aktivitäten anderer Nutzer:innen an.	I look at other users’ (recent) activities.
BV_4	Ich sehe mir die Gruppen anderer Nutzer:innen an.	I look at other users’ groups.
BV_5	Ich schaue, welche Gemeinsamkeiten ich mit anderen Nutzer:innen habe.	I look at what I have in common with other users.
BV_6	Ich folge anderen Nutzer:innenprofilen.	I follow other users’ profiles.
II_1	Ich achte auf bevorstehende Geburtstage meiner Kontakte.	I pay attention to my contacts’ upcoming birthdays.
II_2	Ich sende Geburtstagswünsche oder Glückwunschkarten, die von LinkedIn vorgeschlagen werden.	I send birthday wishes or greeting cards suggested by LinkedIn.
II_3	Ich lese Artikel auf der Startseite.	I read articles on the homepage.
II_4	Ich schreibe und/oder lese private Nachrichten.	I write and/or read private messages.
BI_1	Ich sehe mir Unternehmensprofile an.	I look at company profiles.
BI_2	Ich schaue mir meine Profil-Statistiken an.	I look at my profile statistics.
KA_1	Ich suche nach Jobs (reguläre Suchfunktion; Betrachtung von Jobempfehlungen).	I search for jobs (using the regular search function and viewing job recommendations).
KA_2	Ich organisiere meine Jobsuche (Gespeichert, in Bearbeitung, Beworben, Archiviert).	I organize my job search (saved, in progress, applied, archived).
KA_3	Ich nutze meine Daten von LinkedIn für meine Bewerbungen.	I use my LinkedIn data for my job applications.
SD_1	Ich sehe mir meine eigenen Profildetails an.	I look at my own profile details.
SD_2	Ich überprüfe, ob mein Profilbild mich im besten Licht präsentiert und bearbeite es, falls nötig.	I check whether my profile picture presents me in the best possible light and edit it if necessary.
SD_3	Ich suche nach Personen, die ich kenne oder kennen könnte.	I search for people I know or might know.
SD_4	Ich sehe mir die Profile meiner Profilbesucher:innen an.	I look at the profiles of people who viewed my profile.

*Note*. Based on our exploratory factor analyses, we used as passive factor: BV_1, BV_2, BV_3, BV_4, BV_5, BV_6, BI_1, BI_2, II_3, SD_3, SD_4.
